# Feasibility and User Experience of an AI-Supported mHealth Intervention for Remote Life Goal Setting Based on Flow Theory: Exploratory Within-Participant Study

**DOI:** 10.2196/78717

**Published:** 2026-01-27

**Authors:** Ippei Yoshida

**Affiliations:** 1 Department of Occupational Therapy Faculty of Health Sciences Wakayama Professional University of Rehabilitation Wakayama Japan

**Keywords:** mobile health, mHealth, artificial intelligence, AI, self-management, motivation, occupational therapy, telemedicine, health behavior

## Abstract

**Background:**

Life goal setting contributes substantially to well-being and quality of life, particularly among middle-aged and older adults. However, delivering remote goal-setting support remains challenging due to limited professional resources and accessibility barriers. Recent advancements in mobile health (mHealth) technologies, telemedicine, and generative artificial intelligence (AI) present new opportunities for scalable, personalized health behavior interventions. Nevertheless, few studies have compared AI-driven life goal interventions with conventional human-facilitated approaches in real-world settings.

**Objective:**

This study aimed to evaluate the feasibility and user experience of an AI-supported mHealth intervention for remote life goal setting based on flow theory. We compared the AI-supported approach to occupational therapist (OT)–facilitated support and explored the differential characteristics of AI-guided and human-guided interventions for self-management and motivation enhancement.

**Methods:**

An exploratory, within-participant, 2-condition comparison with a counterbalanced order was conducted among 28 community-dwelling adults (aged between 20 and 76 years) who were smartphone users. Each participant selected 2 personal life goals and completed remote adjusting the challenge-skill balance (R-ACS) sessions, a structured telemedicine process based on flow theory. One goal was supported by an OT, while the other was facilitated by a generative AI chatbot integrated into an mHealth platform. Following each session, participants completed a 4-item rubric-based questionnaire (5-point Likert scale), assessing the quantity and quality of questions, appropriateness of suggestions, and perceived contribution to goal attainment. Free-text feedback was also collected. Quantitative data were analyzed using Wilcoxon signed-rank tests with effect size calculations and Benjamini-Hochberg correction for multiple comparisons. Qualitative differences were explored using text mining (term frequency–inverse document frequency analysis) and sentiment evaluation.

**Results:**

Both AI-supported and OT-facilitated R-ACS sessions were feasible and successfully delivered tailored suggestions for all participants. AI-supported sessions received higher scores on all rubric items than OT-facilitated sessions, with a statistically significant difference in suggestion appropriateness (*z* score=3.13; *P*=.002; *r*=0.418; false discovery rate–adjusted *P*=.008). Term frequency–inverse document frequency analysis of free-text comments revealed that AI-supported sessions emphasized actionability, motivation, and immediacy, while OT-facilitated sessions highlighted reflection, self-understanding, and emotional safety. Participants expressed high acceptance of both intervention types, with AI-supported interactions perceived as particularly accessible and conducive to health behavior change.

**Conclusions:**

AI-supported mHealth interventions for remote life goal setting based on flow theory are feasible, well accepted, and offer potential advantages in immediacy, motivation enhancement, and action-oriented support. OT-facilitated support provides complementary strengths by fostering reflection and psychological safety. A hybrid R-ACS model that integrates both AI and human expertise may optimize personalized, scalable self-management support for life goal setting. Future randomized controlled trials are warranted to further investigate the long-term impact of AI-driven mHealth interventions on health behavior, well-being, and quality of life.

## Introduction

Maintaining and improving the quality of life (QOL) among middle-aged and older adults is a critical issue in an aging society. In particular, having a sense of purpose and life goals has been reported to positively affect the physical and mental well-being of older adults [1-3]. For example, a cohort study by Goto et al [4] showed that older adults without a sense of purpose had significantly higher mortality risks than those with a sense of purpose, and having a sense of purpose also reduced the risk of future physical function decline. Thus, having life goals and a sense of purpose is closely associated with healthy longevity and QOL in middle-aged and older adults [4-6]. Against this background, comprehensive programs that address physical and psychosocial aspects have been explored as suitable interventions to prevent functional decline and improve QOL in older adults [7-9].

Currently, most preventive care programs focus on individual elements, such as exercise, nutrition, and cognitive function, and often combine these elements. However, these approaches make it difficult to comprehensively address the diverse life contexts of older adults and provide personalized support, and their effectiveness requires further examination. In this regard, occupational therapy emphasizes promoting participation in meaningful daily activities as a way to enhance health and well-being. For example, the Lifestyle Redesign program developed in the United States demonstrated significant effects on physical and mental health, occupational function, and life satisfaction among older adults through large-scale randomized controlled trials [10]. The success of Lifestyle Redesign suggests that enhancing engagement in activities and fostering a sense of purpose in life can contribute to older adults’ health. In Japan, Yuri et al [11] conducted an intervention in which life goal–setting support was added to conventional exercise-based preventive care programs for community-dwelling frail older adults and found that the group receiving life goal support showed significantly greater improvements. The study demonstrates that life goal support is a promising intervention for preventing frailty progression and maintaining independence among older adults.

Thus, preventive approaches incorporating life goal–setting support have been suggested to promote not only physical and mental health but also motivate older adults to actively engage in activities. To advance such initiatives further, it is essential to focus on the psychological processes that motivate older adults to engage in health-promoting behaviors. Flow theory [12,13] provides an effective framework for this purpose. Flow theory describes a psychological state in which individuals are deeply immersed in an activity and experience an optimal state in which time seems to fly. This state occurs when perceived challenges and skills are balanced. Flow experience enhances intrinsic motivation, well-being, and self-efficacy [13,14]. Studies in occupational therapy have also emphasized the importance of flow in daily activities for promoting health and quality of life [15-19]. In recent years, such approaches have been increasingly incorporated into digital health and mobile health (mHealth) interventions aimed at behavior change.

The authors also developed the adjusting the challenge-skill balance (ACS) process [20], which evaluates and adjusts the balance between perceived challenges and skills in relation to activities and goals, and reported its effectiveness [20-23]. These findings suggest that ACS based on flow theory can reduce negative experiences during activities and improve QOL among older adults. However, among participants who underwent the ACS process, QOL improvements tended to decline after the follow-up period [23], indicating that the effects may have been limited to direct intervention. Therefore, it is necessary to develop support methods that promote ongoing engagement in life tasks and goals and enhance health literacy and self-management among older adults [24].

To deliver such preventive interventions, the use of information and communication technology, including mHealth and telemedicine approaches, has attracted attention. In recent years, the potential of remote rehabilitation using information and communication technology has been highlighted, and the effectiveness of online interventions in physical therapy has been demonstrated [25-27]. The importance of remote approaches in providing community-based support for older adults is increasing, particularly considering workforce shortages and geographic barriers [28,29]. However, reports on remote interventions in occupational therapy remain limited [30], and few studies have explored artificial intelligence (AI)–driven remote interventions to support life goals or foster a sense of purpose among older adults.

In addition, the use of generative AI for conversational support is gaining attention in psychological support, rehabilitation, and mHealth interventions, with increasing evidence of its effectiveness in health management and mental health [31,32]. For example, AI-based personalized counseling and automated coaching technologies have been suggested to effectively manage stress and improve mental health [33-35]. Furthermore, AI-based digital therapies have been shown to contribute to reducing anxiety and depressive symptoms and may offer new options for remote psychological support [36].

Although remote rehabilitation and generative AI hold promise, it remains unclear whether the existing ACS process (a life goal–support process based on flow theory) can be correctly automated and delivered via AI-supported mHealth interventions. Traditionally, the ACS process involves occupational therapy professionals evaluating the challenge and skill levels related to life tasks and integrating the client’s perceptions with the therapist’s perspective to support appropriate goal setting and adjustment—a process requiring individual adaptation [20,21,23]. In contrast, generative AI provides scalable support by learning patterns from input data and providing guidance based on predetermined algorithms, raising questions about whether it can match the adaptability of human experts. On the other hand, thoughtfully distributing roles between AI and human experts may enable the development of hybrid support models that combine the consistency and immediacy of AI with the flexible judgment and emotional support of professionals.

In domains such as life goal support, which are heavily influenced by personal values and context, exploring hybrid models that leverage the strengths of both AI and human experts is a significant challenge. In this study, we defined *life goals* broadly to encompass not only long-term aspirations but also meaningful daily activities (occupations) that constitute one’s sense of purpose, such as household tasks, hobbies, and social interactions. Although the ultimate target population for this intervention is older adults who require preventive care or rehabilitation, this preliminary feasibility study included a broader age range of adults (age groups: 20-29 and 70-79 years). This was done to verify the usability and interaction quality of the generative AI system across varying levels of digital literacy before narrowing the focus to specific clinical populations.

Therefore, this exploratory study examined the feasibility and appropriateness of remotely delivering ACS through AI-supported mHealth interventions and human-facilitated occupational therapy. Specifically, we compared (1) remote life goal support provided by occupational therapy professionals and (2) AI-supported remote life goal support and explored the differences and usefulness of these approaches. The significance of this study lies in empirically examining the feasibility of remote ACS interventions and the potential for integrating generative AI into scalable mHealth platforms to establish new models for remote health behavior support. Furthermore, by gaining exploratory insights into the characteristics and role-sharing possibilities of AI and human experts, this study aims to provide foundational information for developing practical hybrid mHealth interventions that promote life goal attainment, self-management, and well-being among older adults.

## Methods

### Participants

Participants were 28 community-dwelling adults who owned smartphones, reflecting the target population for mHealth interventions. Participants were recruited through flyers distributed at community centers and social media advertisements. The inclusion criteria included being an adult, residing in the community, owning a smartphone, and using it for daily communication. The sample included 5 (18%) participants in their twenties, 6 (21%) in their thirties, 4 (14%) in their forties, 3 (11%) in their fifties, 5 (18%) in their sixties, and 5 (18%) in their seventies. Basic demographic information, including age and occupation, was also collected (Table 1).

**Table 1:** Participant characteristics (N=28).

Characteristics			Participants (n=28), n (%)
**Age group (y)**			
	20-29	5 (18)	
	30-39	6 (21)	
	40-49	4 (14)	
	50-59	3 (11)	
	60-69	5 (18)	
	70-79	5 (18)	
**Occupation**			
	Company employee	14 (50)	
	Housemaker	5 (18)	
	Self-employed	4 (14)	
	Student	2 (7)	
	Unemployed	3 (11)	

### Procedure

On the basis of the previous studies [20,23], we adopted the ACS process for remote delivery, termed remote ACS (R-ACS). The R-ACS comprises the following structured steps:

1. Goal setting and goal elaboration through initial questions
2. Participant ratings of challenge and skill levels (each on a 7-point scale), along with free-text explanations of their ratings
3. AI-provided or therapist-provided assessment of the participant’s challenge-skill (CS) balance, feedback, and proposal of an adjustment task based on flow theory’s 4-channel model (flow, anxiety, boredom, and apathy)

The participants completed 2 R-ACS sessions, 1 with an occupational therapist (OT) and 1 with AI support, following a within-participant, 2-condition exploratory comparison design (no washout period was implemented). Because the intervention focused on *goal setting* for 2 distinct, independent life goals rather than a physiological treatment where carryover effects are biological, a washout period was deemed unnecessary. Although a learning effect regarding the R-ACS process was possible, the order of intervention (AI first vs OT first) was randomly assigned using a computer-generated randomization sequence to counterbalance this bias across the cohort.

For the OT-supported session, participants engaged in synchronous texting with an experienced OT. To ensure consistency and comparability with the AI condition, the OT followed a strict, structured guide adhering to the same R-ACS steps (steps 1-3) described previously. The therapist used a standardized script for the initial questions and feedback structure, ensuring that the intervention focused solely on the flow theory–based framework without providing extraneous clinical therapy or counseling. Of the 28 participants, 14 (50%) received OT support first, followed by AI support, whereas the remaining 14 (50%) followed the reverse order. Participants were asked to set 2 distinct life goals (1 per session); which goal was addressed under the AI-supported versus OT-supported session depended on the randomized order. Therefore, goal domains were not controlled to be equivalent across conditions; we report the goal category distribution per condition descriptively (Table 2) and address this as a limitation.

**Table 2:** Goal category distribution by intervention condition.

Goal category^a^	Artificial intelligence–supported goals (n=28), n (%)	Occupational therapist–supported goals (n=28), n (%)	Total goals (N=56), n (%)
Work	4 (14)	7 (25)	11 (20)
Health	5 (18)	6 (21)	11 (20)
Hobbies	8 (28)	3 (11)	11 (20)
Family interaction	4 (14)	3 (11)	7 (12)
Lifestyle habits	4 (14)	1 (4)	5 (9)
Childcare	1 (4)	4 (14)	5 (9)
Household tasks	1 (4)	2 (7)	3 (5)
Interaction with friends	1 (4)	2 (7)	3 (5)

^a^Each participant contributed 1 goal per condition (in total 28 goals per condition).

### AI-Supported mHealth Intervention

The AI-supported intervention was implemented using a customized GPT based on GPT-4o (OpenAI), designed specifically for the R-ACS process and integrated into an mHealth delivery format.

### Intervention Flow

The AI guided participants through the following structured steps: (1) goal registration and elaboration (clarifying the goal), (2) CS ratings and rationale (collecting self-rated challenge and skill levels and reasons), (3) independent CS balance estimation (AI estimating the CS balance using the person-environment-occupation model—a theoretical framework commonly applied in occupational therapy [37]), and (4) feedback and adjustment (providing adjustment task suggestions based on flow theory). The AI displayed both user-assessed and AI-assessed CS ratings, prompting reflective dialogue when discrepancies were noted.

### Prompt Structure and Safety

To ensure consistency and safety, the AI’s behavior was governed by a strictly defined system prompt comprising the components mentioned subsequently.

The first component was fixed vs variable elements. The fixed elements included the intervention structure (R-ACS steps), the evaluation logic based on the person-environment-occupation model and flow theory, and the persona (supportive coach tone). The variable elements were limited to the specific content of feedback and goal adjustment suggestions, which the AI generated dynamically in response to each participant’s unique inputs.

The second component was safety rules. Strict safety protocols were implemented within the prompt, including (1) content safety (whereby the AI was explicitly prohibited from providing medical diagnoses or clinical advice), (2) system integrity (with internal instructions and technical specifications, eg, spreadsheet links were concealed to prevent prompt leakage), and (3) privacy protection (whereby the system was instructed to interact using only nicknames and IDs).

The third component was data handling. The system transmitted and stored only the following deidentified data points: date, user ID, nickname, goal content, goal number, user-rated challenge and skill levels and reasons, AI-rated challenge and skill levels and reasons, and the AI’s suggested goal adjustment. No personally identifiable information, such as real names or contact details, was sent or stored.

To minimize potential presentation bias, the researchers reformatted the AI-generated outputs to match the standardized format used in OT-supported interventions. A detailed overview of the prompt structure and constraints is provided in Multimedia Appendix 1.

### Outcome Measures

Feasibility was formally assessed based on the completion rate of the R-ACS sessions. User acceptance and perceived quality were evaluated using the 4-item rubric. After each R-ACS session (OT supported and AI supported), participants completed a 4-item rubric-based questionnaire (5-point scale) to evaluate (1) the amount of questioning, (2) the quality of questions, (3) the appropriateness of suggestions, and (4) the perceived contribution to goal attainment. Free-text comments regarding participants’ experiences were also collected. The rubric was adapted from previous research on coaching skill evaluation [38].

Additionally, this study aimed to explore differences in the characteristics and potential role complementarities of AI-supported and OT-supported interventions in the context of mHealth-based goal support.

### Data Analysis

For the 4 rubric items, within-participant differences (AI vs OT) were analyzed using the Wilcoxon signed-rank test, accounting for the paired nature of the within-participant, 2-condition design. The false discovery rate (FDR) was controlled using the Benjamini-Hochberg method. Effect sizes were calculated as *r*, and bootstrapped 95% CIs were estimated to assess the precision of the effect sizes. Descriptive statistics are reported as medians (IQRs) and means (SDs).

For qualitative analysis, term frequency–inverse document frequency (TF-IDF) analysis was performed to identify characteristic words in participants’ free-text responses across the 2 intervention types. Sentiment analysis was also conducted to assess trends in positive and negative language, following common approaches in mHealth research.

### Ethical Considerations

This study was approved by the ethics committee of Wakayama Professional University of Rehabilitation (WRPUR2024R004). All participants provided informed consent via the mHealth platform before participation. The privacy of participants was strictly protected; all data were deidentified and stored securely, and no personally identifiable information was included in the analysis or reporting. Participants received no financial compensation for their participation. This study was conducted in accordance with the Declaration of Helsinki and ethical guidelines for medical and health research involving human participants.

## Results

### Participants and Procedure

A within-participant, counterbalanced-order 2-condition exploratory comparison was conducted in which 14 (50%) of the 28 participants received OT-supported interventions followed by AI-supported interventions, and 14 (50%) participants received interventions in the reverse order. Each participant set 2 life goals and underwent the R-ACS process for one goal with OT support and the other with AI support, resulting in 56 life goal–setting cases.

Goal categories for 56 goals included work (n=11, 20%), health (n=11, 20%), hobbies (n=11, 20%), family interaction (n=7, 12%), lifestyle habits (n=5, 9%), childcare (n=5, 9%), household tasks (n=3, 5%), and interaction with friends (n=3, 5%). Table 2 shows the goal category distribution for each condition. Because goal domains were not matched, these distributions are reported for transparency. Although the frequency of specific categories, such as hobbies, varied, both interventions covered a broad range of life domains.

### Quantitative Results

Rubric-based questionnaire results indicated that AI-supported interventions generally yielded scores higher than or comparable to OT-supported interventions. Detailed descriptive statistics, including medians and IQRs, are summarized in Table 3.

The Wilcoxon signed-rank test revealed a significant difference in the appropriateness of suggestions (*z* score=3.13; *P*=.002; FDR-adjusted *P*=.008). Specifically, the median score for AI support was 5.0 (IQR 4.0-5.0), which was substantially higher than the 4.0 (IQR 3.0-4.0) observed for OT support. The effect size (*r*) was 0.418 (95% CI 0.16-0.63), indicating a moderate effect. No statistically significant differences were observed for the other items after FDR correction. However, a small-to-moderate effect size was noted for the quality of questions (*r*=0.233).

**Table 3:** Comparison of artificial intelligence (AI)–supported and occupational therapist (OT)–supported interventions—rubric-based questionnaire results.

Outcome item		AI support (n=28)	OT support (n=28)	*P* value^a^		False discovery rate–adjusted *P* value		Effect size^a^, *r* (95% CI)	
**Amount of questioning**					.87		.87		0.021 (–0.24 to 0.29)
	Median (IQR)	3.5 (3.0-5.0)	4.0 (3.0-4.0)						
	Mean (SD)	3.64 (1.13)	3.61 (0.92)						
**Quality of questions**					.08		.11		0.233 (–0.04 to 0.49)
	Median (IQR)	5.0 (4.0-5.0)	5.0 (3.0-5.0)						
	Mean (SD)	4.64 (0.62)	4.29 (0.98)						
**Appropriateness of suggestions**					.002		.008		0.418 (0.16 to 0.63)
	Median (IQR)	5.0 (4.0-5.0)	4.0 (3.0-4.0)						
	Mean (SD)	4.32 (0.94)	3.64 (0.78)						
**Perceived contribution**					.19		.27		0.177 (–0.09 to 0.43)
	Median (IQR)	4.0 (3.8-4.0)	3.0 (3.0-4.0)						
	Mean (SD)	3.89 (0.63)	3.64 (0.78)						

^a^*P* values and effect sizes were calculated using the Wilcoxon signed-rank test.

### Qualitative Results

**Figure 1:**
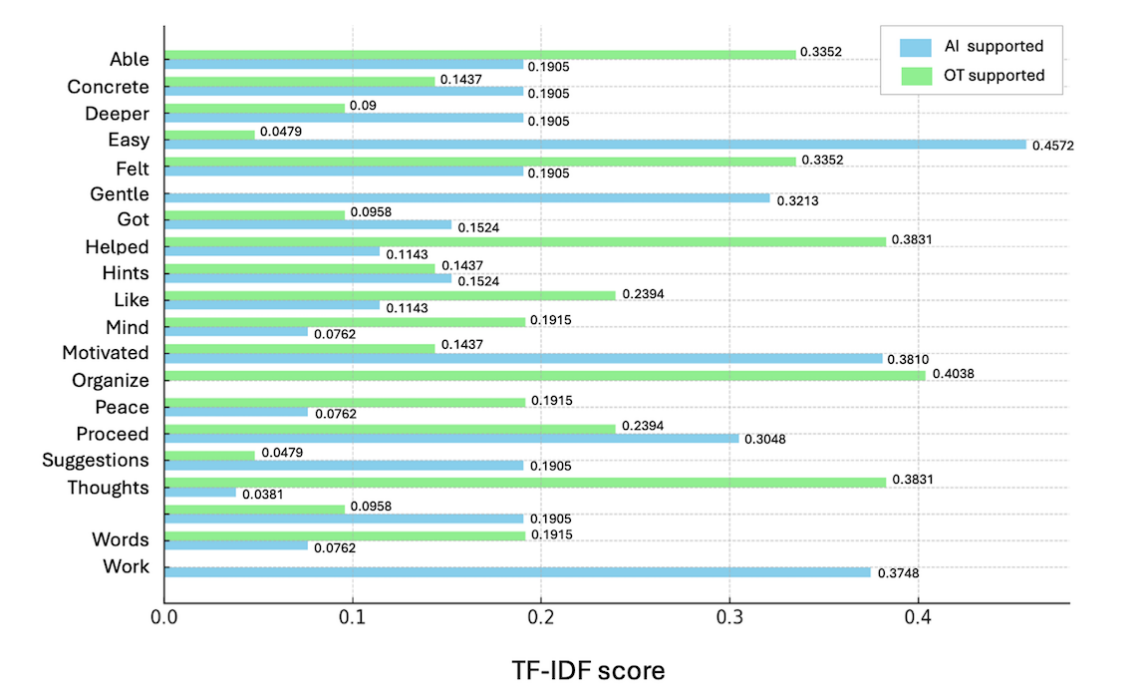
Top characteristic terms identified through term frequency–inverse document frequency (TF-IDF) analysis of free-text responses in artificial intelligence (AI)–supported and occupational therapist (OT)–supported interventions. The bars represent TF-IDF scores, highlighting the terms characteristic of each intervention type.

The TF-IDF analysis of free-text responses revealed distinct lexical patterns between the AI-supported and OT-supported interventions (Table 4). As summarized in Figure 1 and Table 4, AI-supported responses frequently included terms related to motivation, clarity of action, and goal visualization, such as “easy to engage”; “provided reassurance through soft expressions”; “increased motivation to take action”; “clear image of goal attainment”; and “received specific hints.” In contrast, OT-supported responses more frequently included expressions reflecting self-understanding, reflection, and emotional safety, such as “helped verbalize my thoughts”; “assisted in organizing my thinking”; “felt safe while proceeding with the conversation”; and “impressed by the empathetic attitude.”

These results suggest distinct differences in linguistic tendencies and user experiences between the AI-supported mHealth intervention and OT-facilitated goal support.

**Table 4:** Term frequency–inverse document frequency (TF-IDF) scores for top characteristic terms identified from free-text responses.

Term	Artificial intelligence–supported TF-IDF score	Occupational therapist–supported TF-IDF score
Able	0.1905	0.3352
Concrete	0.1905	0.1437
Deeper	0.1905	0.0958
Easy	0.4572	0.0479
Felt	0.1905	0.3352
Gentle	0.3213	0.0000
Got	0.1524	0.0958
Helped	0.1143	0.3831
Hints	0.1524	0.1437
Like	0.1143	0.2394
Mind	0.0762	0.1915
Motivated	0.3810	0.1437
Organize	0.0000	0.4038
Peace	0.0762	0.1915
Proceed	0.3048	0.2394
Suggestions	0.1905	0.0479
Thoughts	0.0381	0.3831
Understanding	0.1905	0.0958
Words	0.0762	0.1915
Work	0.3748	0.0000

## Discussion

### Principal Findings

This study explored the feasibility and characteristics of remote life goal support based on flow theory by comparing OT-supported and AI-supported interventions delivered via mHealth format. The findings showed that AI-supported interventions were rated as equal to or better than OT-supported interventions across all evaluation items, with a statistically significant difference and moderate effect size in the quality of suggestions. Participants also valued the specificity, immediacy, and appropriateness of AI-generated questions and suggestions. This study evaluated the AI intervention as a stand-alone module to isolate its specific characteristics. However, we envision the clinical application of this tool not as a replacement for comprehensive care but as an adjunctive self-management module that could be integrated into broader preventive or rehabilitative programs.

These results suggest that AI can deliver highly structured and effective support within interventional processes such as the ACS, where standardized interaction sequences are beneficial for promoting self-management in remote settings.

Furthermore, to ensure a fair comparison, AI-generated outputs were reformatted by the researchers and presented to participants in a standardized format comparable to OT support, thereby minimizing potential biases related to presentation style. Therefore, the observed differences are likely attributable to the inherent characteristics of the interventions rather than formatting effects.

### Comparison With Prior Work

These findings align with the growing body of evidence supporting the effectiveness of generative AI–based psychological and behavior change interventions within digital health. A recent randomized controlled trial [39] demonstrated that a generative AI chatbot significantly improved mental health outcomes. Similarly, in this study, AI-supported mHealth interventions demonstrated strengths in promoting user engagement and facilitating actionable life goal pursuit.

In addition, we conducted a TF-IDF analysis of free-text comments as an appropriate exploratory method to extract characteristic lexical patterns from participants’ subjective feedback systematically. This analysis provided complementary insights into participants’ perceptions and experiences of each form of support, enriching the understanding of intervention-specific characteristics.

Our results also correspond with findings from Ref-Layers, an AI-based reflection system that enhances users’ metacognitive strategy use through structured dialogue [40]. The AI-supported presentation of CS balance and the guided reconsideration of reasoning in this study may have similarly contributed to participants’ self-understanding and reflective thinking.

Moreover, AI-driven interactions may help reduce interpersonal anxiety and promote self-disclosure, thereby facilitating more proactive engagement in health behavior change and goal setting. Previous research [31,32] suggests that users perceive virtual agents as more confidential and less judgmental than their human counterparts, encouraging openness. Consistent with these findings, other studies [41] have reported that interactions with virtual agents can enhance perceived privacy and promote candid self-disclosure.

The immediacy of AI responses emerged as a notable advantage, particularly in remote mHealth interventions where real-time dialogue is essential. Previous studies [42,43] have reported that fast and consistent chatbot responses enhance user satisfaction and engagement. In contrast, while OT-supported interventions provide valuable, nuanced, and context-sensitive feedback, they inherently involve longer response times.

### Limitations

This study has several limitations. First, participants’ previous beliefs and expectations regarding AI may have influenced their evaluations. Additionally, we did not quantitatively assess participants *AI literacy* or previous familiarity with generative AI tools. Users with higher AI literacy might have navigated the AI prompts more effectively, potentially skewing satisfaction scores. Previous research has shown that priming users’ beliefs about AI can alter perceptions of trustworthiness, empathy, and effectiveness [44]. Future studies should incorporate strategies to control for these effects.

Second, we did not quantitatively measure the duration of each session. However, participant feedback suggested a perception of immediacy in the AI interaction compared to the OT support.

Third, this study evaluated only a single intervention session, thereby limiting the ability to assess the long-term sustainability of AI-supported mHealth interventions. However, emerging evidence suggests that AI-based coaching can maintain its effectiveness over extended periods. For example, Terblanche et al [45] reported that AI coaching supported clients’ goal attainment over a 10-month period with efficacy comparable to that of human coaching. Nevertheless, rigorous evaluation of long-term effects within life goal interventions remains essential.

Fourth, the relatively small sample size and wide age range may limit the generalizability of these findings. Future studies should examine variations in acceptance and effectiveness across different life stages and demographic groups to further validate the intervention. In addition, although the AI model used in this study effectively implemented a structured ACS process, it currently lacks the capability to fully adapt to individual users or interpret subtle psychological states and nonverbal cues—areas where human-facilitated support remains superior. Further advancements in natural language processing and integration of multimodal data are needed to enhance the adaptability of AI-supported mHealth interventions.

Finally, several methodological limitations regarding the study design and authorship must be acknowledged. Because participants set different life goals for the AI and OT conditions, and goals were not matched for domain or perceived difficulty, the observed between-condition differences may be partly attributable to goal characteristics rather than the support modality alone. To improve transparency, we report the goal category distribution per condition (Table 2); however, we did not formally test distributional differences due to the exploratory design. Future studies should match goal domains across conditions or adjust for goal characteristics (eg, baseline challenge and skill ratings). Additionally, the primary investigator (IY) was involved in both system development and study evaluation. Although financial conflicts of interest were absent, this concentration of roles introduces a potential risk of developer bias. Future large-scale trials should use independent evaluators and randomized goal allocation to mitigate these biases.

### Conclusions and Future Directions

This study clarified the distinct strengths and limitations of AI-supported and OT-supported ACS interventions delivered via remote formats. The ability of AI to deliver structured, consistent, and immediate support makes it a promising model for scalable mHealth interventions, particularly in settings with limited human resources or geographic barriers. Conversely, personalized adaptation, emotional support, and nuanced judgment provided by occupational therapy remain indispensable in contexts where human-facilitated care is essential.

The development and evaluation of a hybrid R-ACS model that integrates the complementary strengths of both approaches is warranted. One potential model could involve OT involvement during the initial goal-setting and assessment phases, followed by AI-supported mHealth interventions providing ongoing self-monitoring, feedback, and behavior change support. Periodic OT interactions could be used to reassess and recalibrate goals and strategies, ensuring both adaptability and intervention consistency.

Such hybrid approaches may maximize the benefits of AI and human support, leading to more effective, scalable, and sustainable remote interventions for promoting life goal attainment, self-management, and well-being. Future research, including randomized controlled trials, should rigorously evaluate these hybrid models, explore improvements in AI adaptability and OT-AI collaboration interfaces, and advance the development of innovative digital health services that support personalized goal setting.

Flow theory has also been widely applied to analyze user experiences in digital health interventions, including AI-driven chatbot interactions, as it helps explain user immersion, motivation, and satisfaction [46]. Leveraging this theoretical framework may further enhance the design and effectiveness of AI-supported mHealth interventions aimed at promoting personalized goal setting and health behavior change.

## Appendix

Multimedia Appendix 1 Artificial intelligence system instructions for the remote adjusting the challenge-skill balance process.

## Abbreviations

ACS: adjusting the challenge-skill balance
AI: artificial intelligence
CS: challenge skill
FDR: false discovery rate
mHealth: mobile health
OT: occupational therapist
QOL: quality of life
R-ACS: remote adjusting the challenge-skill balance
TF-IDF: term frequency–inverse document frequency

## Data Availability

The datasets generated or analyzed during this study are not publicly available due to participant privacy protection policies but are available from the corresponding author on reasonable request.
